# The Bruton’s Tyrosine Kinase Inhibitor Ibrutinib Impairs the Vascular Development of Zebrafish Larvae

**DOI:** 10.3389/fphar.2020.625498

**Published:** 2021-01-13

**Authors:** Kun Wang, Qiushi Xu, Hanbing Zhong

**Affiliations:** Department of Biology, Southern University of Science and Technology, Shenzhen, China

**Keywords:** ibrutinib, zebrafish, adverse effects, vascular toxicity, angiogenesis

## Abstract

Ibrutinib is an orally bioavailable, irreversible selective Bruton’s tyrosine kinase inhibitor that has demonstrated impressive therapeutic effects in patients with B cell malignancies. However, adverse effects, such as bleeding and hypertension, are also reported, implying that studies on the toxicological effect of ibrutinib on living organisms are needed. Here, we have used zebrafish, a successful model organism for studying toxicology, to investigate the influence of ibrutinib during embryogenesis. We found that ibrutinib had potent toxicity on embryonic development, especially vascular development in zebrafish embryos. We also revealed that ibrutinib perturbed vascular formation by suppressing angiogenesis, rather than vasculogenesis. In addition, ibrutinib exposure led to the collapse of the vascular lumen, as well as reduced proliferation and enhanced apoptosis of vascular endothelial cells. Moreover, the expression of vascular development-related genes was also altered in ibrutinib-treated embryos. To our knowledge, this is the first study to describe the vascular toxicity of ibrutinib in an animal model, providing a theoretical basis for clinical safety guidelines in ibrutinib treatment.

## Introduction

Bruton’s tyrosine kinase (BTK) is a Tec family tyrosine kinase that plays an essential part in the B cell receptor (BCR) signaling pathway, which is responsible for proliferation, survival, and maturation (Satterthwaite and Witte, 2000; Mohamed et al., 2009; Buggy and Elias, 2012). Ibrutinib is an orally bioavailable, irreversible selective BTK inhibitor, which has demonstrated impressive therapeutic effects in patients with B cell malignancies, such as mantle cell lymphoma and chronic lymphocytic leukemia (Byrd et al., 2013; Wang et al., 2013; Herman et al., 2014; de Rooij et al., 2015). Although ibrutinib is generally well tolerated, toxicities and adverse events have been reported (Paydas, 2019; Lasica and Tam, 2020), including atrial fibrillation (Thompson et al., 2016; Shanafelt et al., 2017), bleeding (Kunk et al., 2016; Caron et al., 2017), hypertension (Dickerson et al., 2019), and infections (Sun et al., 2015). The undesirable side effects of ibrutinib can be partly attributed to the off-target effects caused by non-specific interactions with other kinases. Besides BTK, ibrutinib also binds to proteins that are involved in other important signaling pathways, such as bone marrow expressed kinase (BMX), epidermal growth factor receptor (EGFR), interleukin-2-inducible T cell kinase (ITK), and Janus kinase 3 (JAK3) (Liang et al., 2018; Kim, 2019). Thus, more studies on the toxicological effect of ibrutinib in living organisms are needed to provide clinical guidelines for ibrutinib application.

The formation of the cardiovascular system plays a pivotal role in the development of the vertebrate embryo. During embryogenesis, hemangioblasts give rise to two cell populations, primitive hematopoietic cells, and vascular endothelial precursor cells, which are also called angioblasts (Eilken et al., 2009; Lancrin et al., 2009). The development of the vascular system involves two processes, vasculogenesis and angiogenesis. Vasculogenesis defines the process by which angioblasts form *de novo* blood vessels, while angiogenesis is the formation of new blood vessels sprouting from pre-existing ones (Risau et al., 1988). Several key factors are known to be crucial for vascular formation. Previous studies demonstrate that the vascular endothelial growth factor (VEGF) family and their receptors (VEGFR) are required for both vasculogenesis and angiogenesis (Carmeliet et al., 1996; Ferrara et al., 2003). Vascular development, especially angiogenesis, is closely related to the pathogenesis of several chronic diseases, such as cancer, arthritis, and diabetic retinopathy (Carmeliet, 2003; Carmeliet, 2005). Angiogenesis is an effective target in the therapies of these diseases. On the other hand, the clinical uses of anti-angiogenic agents are associated with the increased risk of vascular toxicities, including bleeding, hypertension, and thrombosis (Kabbinavar et al., 2003; Motzer et al., 2006; Horsley et al., 2012). A recent study suggests that ibrutinib-treated macrophages reduce the tube formation of endothelial cells (ECs) *in vitro* (Ping et al., 2017), and structure-based drug repositioning predicts that ibrutinib may be a VEGFR2 inhibitor (Adasme et al., 2020). However, there is no further evidence or *in vivo* study to confirm whether ibrutinib can influence vascular development.

Zebrafish has been widely used as a model organism in studying embryonic development and the toxicological effects of drugs (Sukardi et al., 2011; MacRae and Peterson, 2015). Furthermore, zebrafish are known to be a successful model for investigating vascular development because the basic framework of vascular anatomy and the molecular mechanisms of vascular formation are conserved between zebrafish and humans (Ellertsdottir et al., 2010; Gore et al., 2012). In the present study, we demonstrate the adverse effects of ibrutinib on vascular development in zebrafish embryos. We reveal that ibrutinib strongly suppresses the sprouting and formation of new vessels and reduces vascular lumen size. In addition, we verify that ibrutinib exposure leads to abated proliferation and excessive apoptosis of vascular endothelial cells (VECs). Moreover, we show that the expression of crucial genes associated with vascular development is altered by ibrutinib. Overall, this study demonstrates that ibrutinib exposure exhibits vascular toxicity during embryogenesis, providing an important basis for the safety guidelines of ibrutinib in clinical application.

## Materials and Methods

### Zebrafish Lines and Maintenance

The zebrafish lines TU and *Tg* (*kdrl:EGFP*) were obtained from the Guangzhou Institutes of Biomedicine and Health, Chinese Academy of Sciences, and maintained at 26–28°C with 14/10 h light/dark cycles in an automatic zebrafish housing system (Haisheng, Shanghai, China) as described previously (Westerfield, 1993). Zebrafish were fed twice daily with live brine shrimp. For embryos collection, one male and one female zebrafish were separated in a spawning box overnight, and spawning was triggered by the light on the next morning. Embryos were collected within 30 min and then maintained at 28.5°C. To inhibit pigmentation, embryos were incubated in embryo medium (5 mM NaCl, 0.17 mM KCl, 0.33 mM CaCl_2_, 0.33 mM MgSO_4_, and 0.002% methylene blue) containing 0.003% phenylthiourea (Sigma-Aldrich, St. Louis, MO, United States).

### Chemical Exposure

Chemicals (ibrutinib and spebrutinib) were purchased from MedChemExpress (Monmouth Junction, NJ, United States). The stock solution (25 mM) was prepared by dissolving in dimethyl sulfoxide (DMSO, Sigma-Aldrich). Embryos at 12 h post-fertilization (hpf) were randomly grouped (20 embryos for each group) into six-well plates containing 4 ml of different concentrations of chemicals (stock solution dissolved in embryo medium) and incubated at 28.5°C. Embryo medium containing 1% DMSO was used as a vehicle control. Chemical solutions were renewed every 12 h.

### Vasculature Observation

To observe vascular development *in vivo*, the transgenic strain *Tg* (*kdrl:EGFP*) was used. Exposed embryos were mounted in 1% low gelling temperature agarose (Sigma-Aldrich) with 0.03% tricaine (Sigma-Aldrich) in a 15-mm glass-bottomed dish, and images were acquired at ×100 magnification using a confocal microscope (TCS SP8, Leica, Wetzlar, Germany). An intersegmental vessel (ISV) that reached the dorsal lateral region was considered normal and the percentage of normal ISVs in each embryo was calculated. The diameters of the dorsal aorta (DA) and posterior cardinal vein (PCV) in exposed embryos were measured at 72 hpf.

### Morpholino Injection

The *btk* MO (sequence: TCC​AGA​ACT​CTG​TCT​GCC​ATG​TCT​A) was ordered from Gene Tools (Philomath, OR, United States) and dissolved in sterile water. Embryos at the one-cell stage were injected with 0.5–1 nL *btk* MO (100 μM) using a microinjector (PLI-100A, Warner Instruments, Holliston, MA, United States). Injected embryos were incubated in embryo medium at 28.5°C, and vasculature was observed at 72 hpf.

### Histological Analysis

Exposed embryos were fixed in 4% paraformaldehyde (PFA, Sigma-Aldrich) for 24 h at 4°C, then dehydrated by ethanol, cleared by xylene, and embedded in paraffin. Tissues were sectioned at 4 μm thickness and stained with hematoxylin and eosin (H&E) (Beyotime, Shanghai, China) according to the manufacturer’s instructions.

### Immunofluorescence Analysis

Exposed embryos were fixed, embedded, and sectioned as described above. Tissue sections were deparaffinized, rehydrated in PBS, and repaired in EDTA antigen retrieval buffers. After blocking by bovine serum albumin (BSA, Sigma-Aldrich), slides were incubated with GFP antibody (rabbit, 1:200, GeneTex, Irvine, CA, United States) overnight at 4°C, and then incubated with goat anti-rabbit IgG antibody (DyLight594, 1:500, GeneTex) at room temperature for 2 h in dark conditions. Additionally, slides were counterstained with DAPI (Sigma-Aldrich) at room temperature for 15 min in dark conditions.

### EdU Assay

Cell proliferation was detected using Click-iT Plus EdU Imaging Kits (Thermo Fisher Scientific, Waltham, MA, United States). Embryos of *Tg(kdrl:EGFP)* were treated with ibrutinib at 12 hpf and transferred into 1 ml EdU (500 μM, dissolving in embryo medium containing 10% DMSO) at 24 hpf. After incubating on ice for 1 h, embryos were transferred back to embryo medium containing ibrutinib and incubated at 28.5°C. Embryos were fixed in 4% PFA at 36 hpf and stained with EdU according to the manufacturer’s instructions. For double staining with VECs, embryos were incubated with GFP antibody (rabbit, 1:100, GeneTex) after blocking by goat serum (Thermo Fisher Scientific), and then incubated with goat anti-rabbit IgG antibody (DyLight594, 1:200, GeneTex).

### Tunel Assay

Cell apoptosis was detected using an *In Situ* Cell Death Detection Kit (Roche, Mannheim, Germany). *Tg* (*kdrl:EGFP*) embryos were treated with ibrutinib at 36 hpf and fixed in 4% PFA at 60 hpf. TUNEL staining was performed according to the manufacturer’s instructions, followed by double staining with VECs as described above.

### Whole-Mount *In Situ* Hybridization (WISH)

RNA probes were obtained from the South China University of Technology. Exposed embryos were fixed in 4% PFA overnight at 4°C, and WISH was performed at a hybridization temperature of 60°C as previously described (Thisse and Thisse, 2008).

### Imaging

Images of H&E and WISH staining were taken under a Leica DMi8 microscope with a Leica DFC450 C camera. Images of immunofluorescence, EdU, and TUNEL staining were acquired using a Leica TCS SP8 confocal microscope.

### Quantitative Real-Time Polymerase Chain Reaction

Total RNA samples were isolated from exposed embryos at 24 and 48 hpf by using TRIzol reagent (Thermo Fisher Scientific), and cDNA was generated with a Maxima H Minus First Strand cDNA Synthesis Kit (Thermo Fisher Scientific) according to the manufacturer’s instructions. Then, qRT-PCR was performed with a PowerUp SYBR Green Master Mix (Applied Biosystems, Foster City, CA, United States) on a StepOnePlus Real-Time PCR System (Applied Biosystems). Each experiment was analyzed in quadruplicate and the fold changes were determined by the ΔΔ comparative threshold method. Primer sequences are shown in Table 1 as previously described (Ningappa et al., 2015; Bower et al., 2017; Lei et al., 2017).

**TABLE 1:** Primer sequences for qRT-PCR.

Gene	GenBank No.	Forward primer (5′→3′)	Reverse primer (5′→3′)
*eef1a*	NM_131263.1	TAC​TTC​TCA​GGC​TGA​CTG​TG	ATC​TTC​TTG​ATG​TAT​GCG​CT
*flt1*	NM_001014829.3	TGA​GAC​CAT​CGT​TGA​TGG​AG	CTG​ATG​GAC​ACC​CTG​GAG​TT
*flt4*	NM_130945.2	AAA​GGG​GAG​ACA​ACG​ACA​TG	CGG​CAC​TAA​CGA​GAA​GAG​AG
*kdr*	NM_001024653.2	ATT​CGT​TCT​TAC​GGG​GGT​TG	CTC​TAT​CGC​TTT​AGC​CAC​GT
*kdrl*	NM_131472.1	CTG​GTG​GAG​AGG​CTA​GGA​GA	TGA​TCG​GGA​TGT​AGT​GCT​TTC
*vegfaa*	NM_131408.3	GAC​GTT​TCG​TGT​CTC​TGT​CG	AAA​AGA​GTG​CGT​GCA​AGA​CC
*vegfab*	NM_001328597.1	GGA​CCT​GCA​GAT​GTG​ACA​AA	ATC​AAA​TCC​TGT​GCT​CCG​AG
*vegfc*	NM_205734.1	GGC​CTC​AAC​AGA​GCT​TCA​AC	TCT​CTT​GGG​GTC​CAC​GTT​AC
*vegfd*	NM_001040178.1	GCT​GGA​CTT​CAC​ATG​TTG​CT	CTC​AGT​TCC​TGC​TCC​CAC​TT
*egfra*	NM_194424.1	GCC​TGA​TCT​AAA​GGA​CTG​CAA​AG	GCC​AGT​AGA​CCT​CCG​ACA​A
*egfrb*	CN171326.1	CAA​ATG​TGA​AGG​CTT​GTG​TCC	GAT​GTT​GGT​TGC​GTT​GAC​TG
*itk*	NM_131104.1	AAC​GGA​GGC​AGA​GGA​CTT​AC	CGC​CAT​TAG​ACC​CTG​AAA​CT
*jak3*	XM_002663087.6	GAG​ATA​CCT​GCG​ATT​CAT​CTC​T	GTG​CTG​TAG​CAG​ATG​CCC​TT

### Statistical Analysis

Data are presented as mean ± SEM. Data were analyzed using the Student’s *t*-test for comparisons between two groups, and one-way ANOVA (followed by Dunnett’s test) for comparisons among multiple groups. Differences were considered significant at *p* < 0.05.

## Results

### Ibrutinib Disturbs the Development of Zebrafish Embryos

**FIGURE 1:**
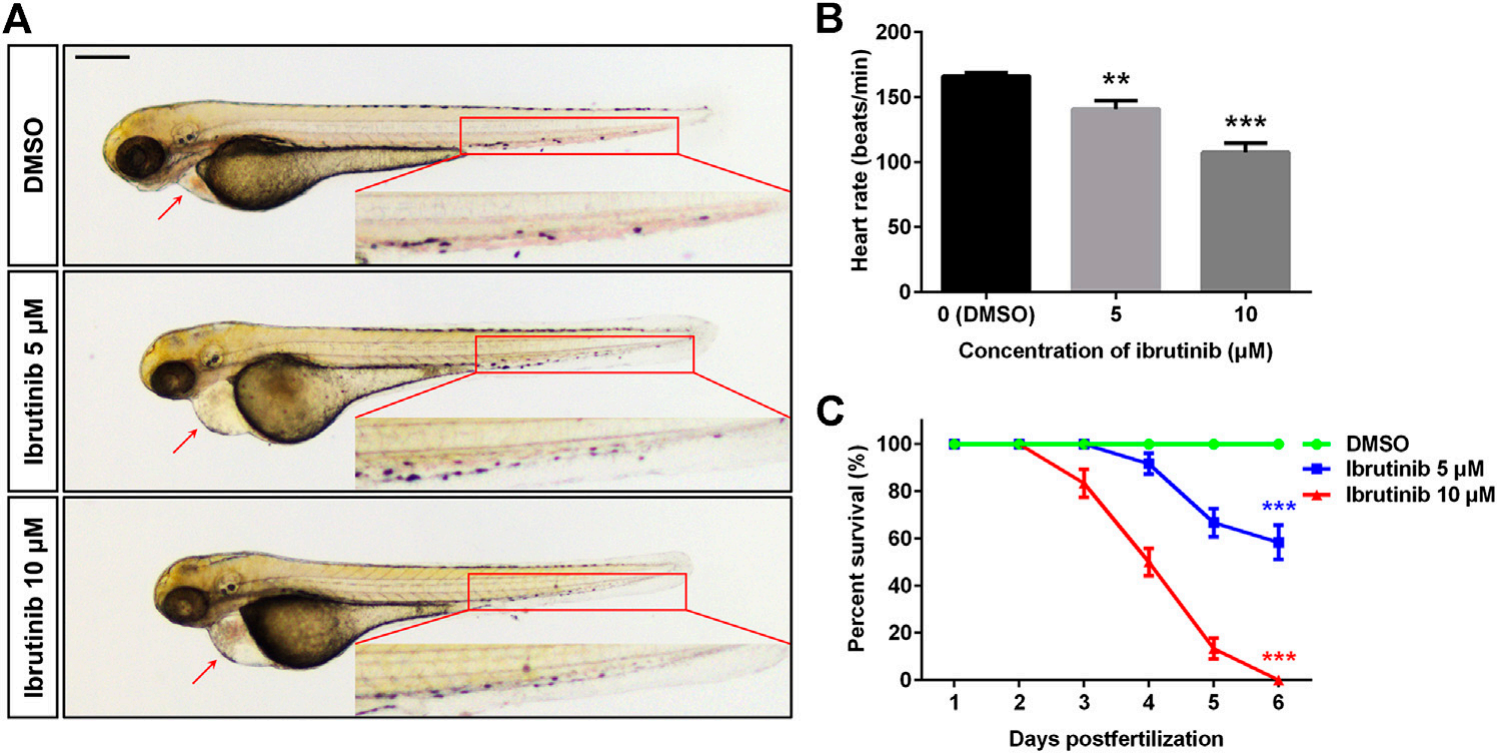
Ibrutinib had potent toxicity in zebrafish embryos. **(A)** Ibrutinib induced a developmental deficiency in treated embryos at 72 h post-fertilization (hpf); a shorter body length, pericardial edema, and arrested blood flow were observed. Scale bar: 300 μm. **(B)** Heart rate of ibrutinib-treated embryos at 72 hpf; *n* = 10 for each group. **(C)** Survival curves of embryos treated with DMSO or ibrutinib at 12 hpf, survival rate was calculated from three independent experiments; *n* = 60 for each group in total. Asterisks indicate significant differences (DMSO vs. ibrutinib), **p < 0.01, ***p < 0.001.

To investigate the effect of ibrutinib on zebrafish during embryogenesis, embryos at 12 hpf were exposed to various concentrations (0, 1, 5, 10, and 20 μM) of ibrutinib. Malformations were observed in embryos exposed to 5 and 10 μM ibrutinib at 72 hpf when compared with embryos treated by DMSO, including shorter body length, pericardial edema, slower or arrested blood flow, and decreased heart rate (Figures 1A,B). About 60% of embryos exposed to 5 μM ibrutinib survived at 6 days post-fertilization (dpf), but all embryos exposed to 10 μM ibrutinib died at the same stage (Figure 1C). Embryos treated by 1 μM of ibrutinib showed no obvious phenotype until 6 dpf, whereas 20 μM of ibrutinib exposure was lethal to all embryos within 12 h after treatment (data not shown). Therefore, these results suggested that ibrutinib had potent toxic effects on embryonic development in zebrafish.

### Ibrutinib Perturbs Vascular Formation in Zebrafish Embryos

**FIGURE 2:**
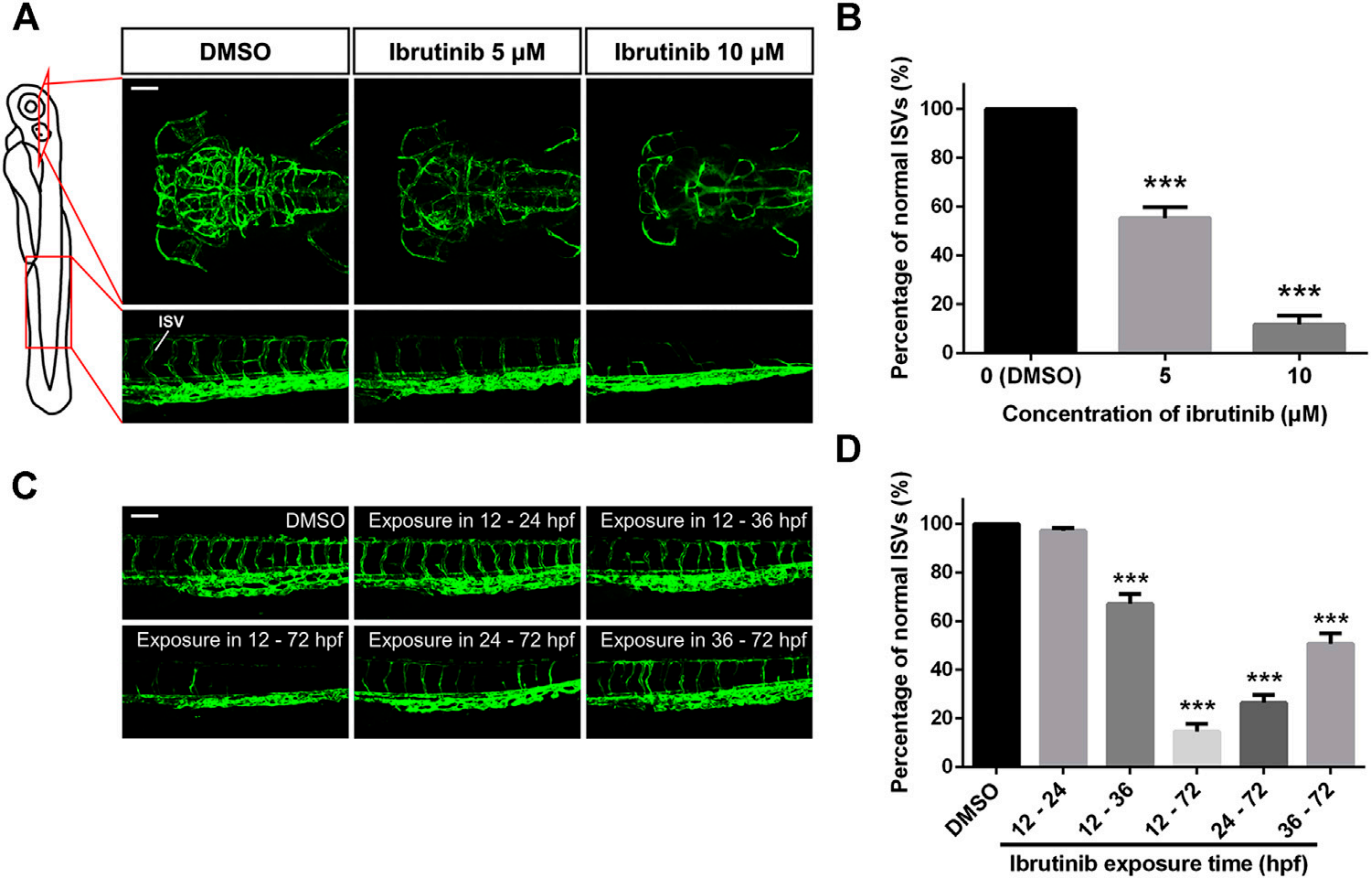
Vascular formation was perturbed in ibrutinib-treated embryos. **(A)** Confocal images of blood vessels in the brain and trunk of ibrutinib-treated *Tg* (*kdrl:EGFP*) embryos at 72 h post-fertilization (hpf). Scale bar: 100 μm. **(B)** The percentage of normal intersegmental vessels (ISVs) in embryos presented at **(A)**; *n* = 10 for each group. **(C)** Confocal images of blood vessels in the trunk of embryos treated and washed at different stages. Scale bar: 100 μm. **(D)** The percentage of normal ISVs in embryos presented at **(C)**; *n* = 10 for each group. Asterisks indicate significant differences (DMSO vs. ibrutinib), ***p < 0.001.

Since blood flow was strongly suppressed in embryos exposed to ibrutinib, we speculated vascular formation of these embryos might be inhibited as well. Thus, the transgenic zebrafish line *Tg* (*kdrl:EGFP*) was used in the direct observation of vascular development in living embryos. Embryos were exposed to 1% DMSO, 5, or 10 μM ibrutinib at 12 hpf and observed by a confocal microscope at 72 hpf. Our results showed that in embryos treated with ibrutinib, the growth of blood vessels in both brain and trunk was dramatically impeded (Figure 2A), and the number of normal ISVs was significantly decreased, compared with embryos treated by DMSO (Figure 2B). To further investigate the inhibition of vascular development by ibrutinib, we treated embryos with 10 μM ibrutinib at various stages (12, 24, and 36 hpf), followed by a wash out at respective stages (24 and 36 hpf, no wash). All treated embryos were observed at 72 hpf, and the number of ISVs was counted. The results indicated that ibrutinib exposure at 12 hpf strongly suppressed ISV growth, and obvious inhibitory effects were also observed at 24 and 36 hpf (Figures 2C,D). However, ISV growth was barely inhibited when ibrutinib was washed out at 24 hpf, or weakly perturbed when ibrutinib was washed out at 36 hpf (Figures 2C,D). These results imply that continuous exposure of ibrutinib was essential for the inhibition of vascular formation.

To verify whether ibrutinib perturbed vascular formation through BTK activity, we used another BTK inhibitor, spebrutinib, as well as *btk* morpholino. *Tg* (*kdrl:EGFP*) embryos were treated with 10 μM spebrutinib at 12 hpf, or were injected with *btk* MO (100 μM, 0.5–1 nl for each embryo) at the one-cell stage. Vascular development was nearly intact in both spebrutinib-treated embryos and morpholino-injected embryos at 72 hpf (Supplementary Figures S1A,B), indicating that BTK inhibition or knockdown would not affect vascular development. Moreover, we performed qRT-PCR to assess the expression of *egfra*, *egfrb*, *itk*, and *jak3* in ibrutinib-treated embryos. The results indicated that these genes were unaffected by ibrutinib in zebrafish (Supplementary Figure S1C).

### Ibrutinib Induces the Reduction of Vascular Lumen Size

**FIGURE 3:**
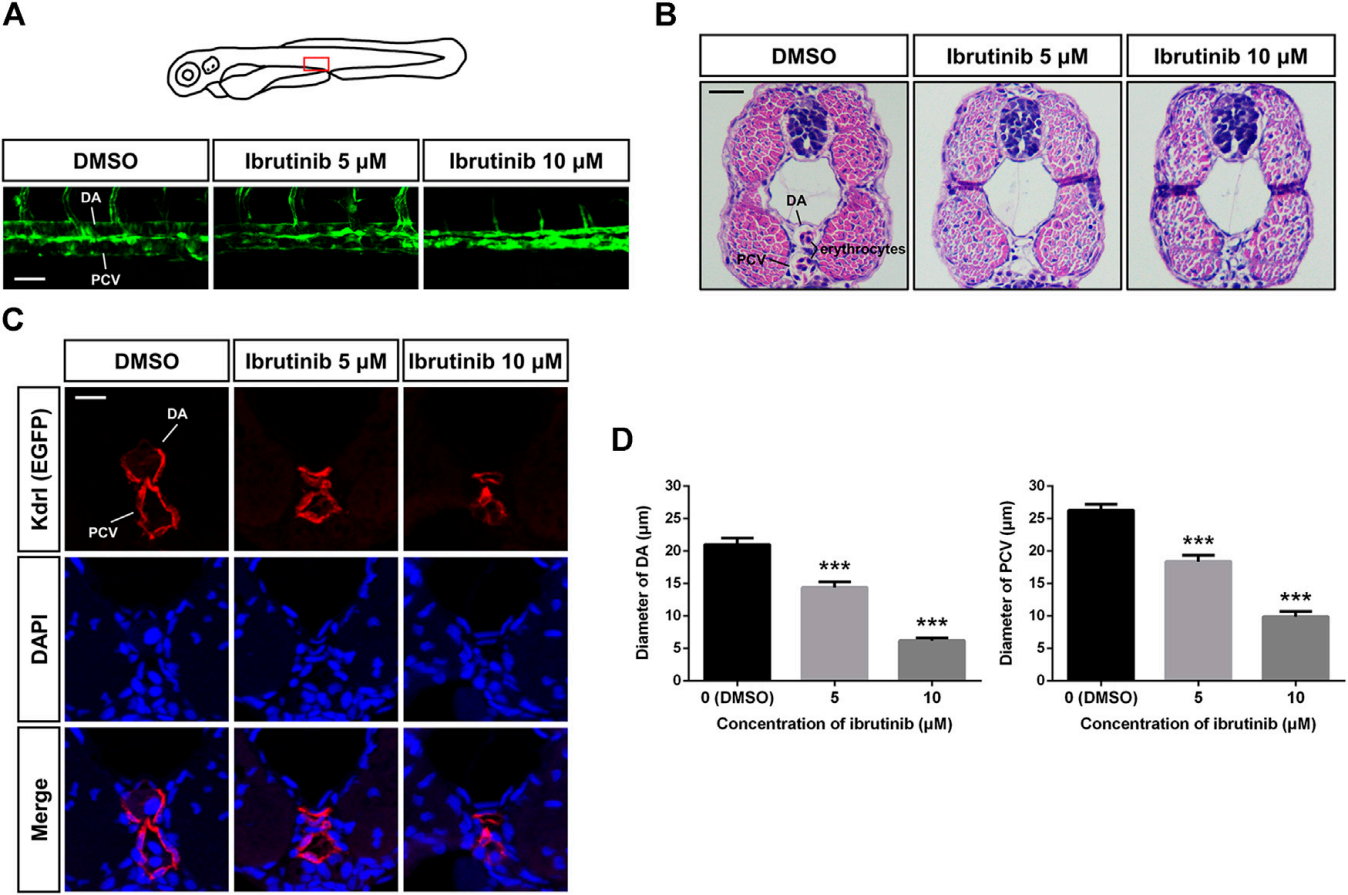
Vascular lumens were collapsed after ibrutinib treatment. **(A)** Confocal images of the dorsal aorta (DA) and posterior cardinal vein (PCV) in the trunk of ibrutinib-treated *Tg* (*kdrl:EGFP*) embryos at 72 h post-fertilization (hpf). Scale bar: 50 μm. **(B)** H&E staining of cross-sections containing the DA and PCV of treated embryos at 72 hpf. Scale bar: 20 μm. **(C)** Immunofluorescence staining of cross-sections containing the DA and PCV of treated embryos at 72 hpf. Scale bar: 10 μm. **(D)** Diameters of the DA and PCV in treated embryos at 72 hpf; *n* = 10 for each group. Asterisks indicate significant differences (DMSO vs. ibrutinib), ***p < 0.001.

Besides the inhibition of newly formed blood vessels, we also noticed that vascular lumen size was reduced in ibrutinib exposed embryos, whereby both the DA and PCV were narrower than the control embryos treated by DMSO (Figure 3A). H&E and immunofluorescence staining of cross-sections further revealed that ECs formed larger vascular lumens containing erythrocytes in control embryos, whereas vascular lumens in ibrutinib-exposed embryos were much smaller and contained no erythrocytes (Figures 3B,C). The diameters of the DA and PCV were measured and confirmed this conclusion (Figure 3D).

### Ibrutinib Exposure Leads to Abated Proliferation and Excessive Apoptosis of VECs

**FIGURE 4:**
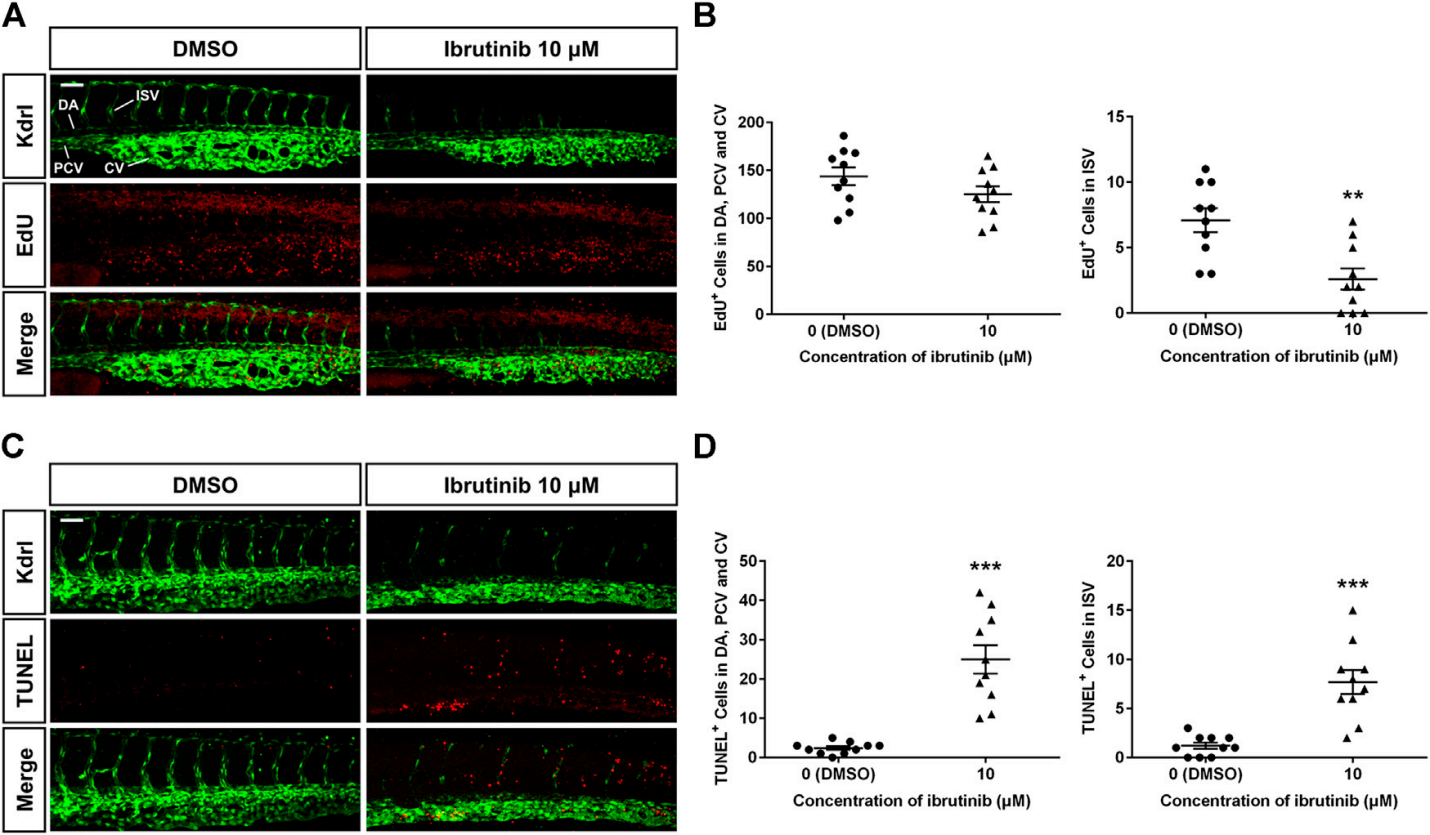
Reduced proliferation and enhanced apoptosis of vascular endothelial cells (VECs) in ibrutinib-treated embryos. **(A)** An EdU assay showed proliferation signals in the VECs of ibrutinib-treated embryos at 36 h post-fertilization (hpf). **(B)** The number of proliferated VECs was counted in the dorsal aorta (DA), posterior cardinal vein (PCV), and caudal vein (CV), as well as ISVs; *n* = 10 for each group. **(C)** A TUNEL assay showed apoptosis signals in the VECs of ibrutinib-treated embryos at 60 hpf. **(D)** The number of apoptotic VECs was counted in the DA, PCV, and CV, as well as intersegmental vessels (ISVs); *n* = 10 for each group. Asterisks indicate significant differences (DMSO vs. ibrutinib), **p < 0.01, ***p < 0.001.

To determine how vascular development was disturbed, we examined the proliferation and apoptosis of VECs, using *Tg* (*kdrl:EGFP*). An EdU assay at 36 hpf showed that proliferation signals in the ISVs of embryos treated by ibrutinib were decreased compared with those in embryos treated by DMSO (Figures 4A,B). In contrast, the number of proliferation signals in the DA, PCV, and caudal vein (CV) was similar in both ibrutinib and DMSO treated embryos (Figures 4A,B). A TUNEL assay at 60 hpf revealed that apoptotic signals on VECs, including ISVs and the DA, PCV, and CV, were significantly increased in embryos treated by ibrutinib compared with embryos treated by DMSO (Figures 4C,D). Collectively, these results suggest that ibrutinib abated proliferation and induced excessive apoptosis of VECs during embryogenesis.

### Ibrutinib Exposure Alters the Expression of Vascular Development-Related Genes

**FIGURE 5:**
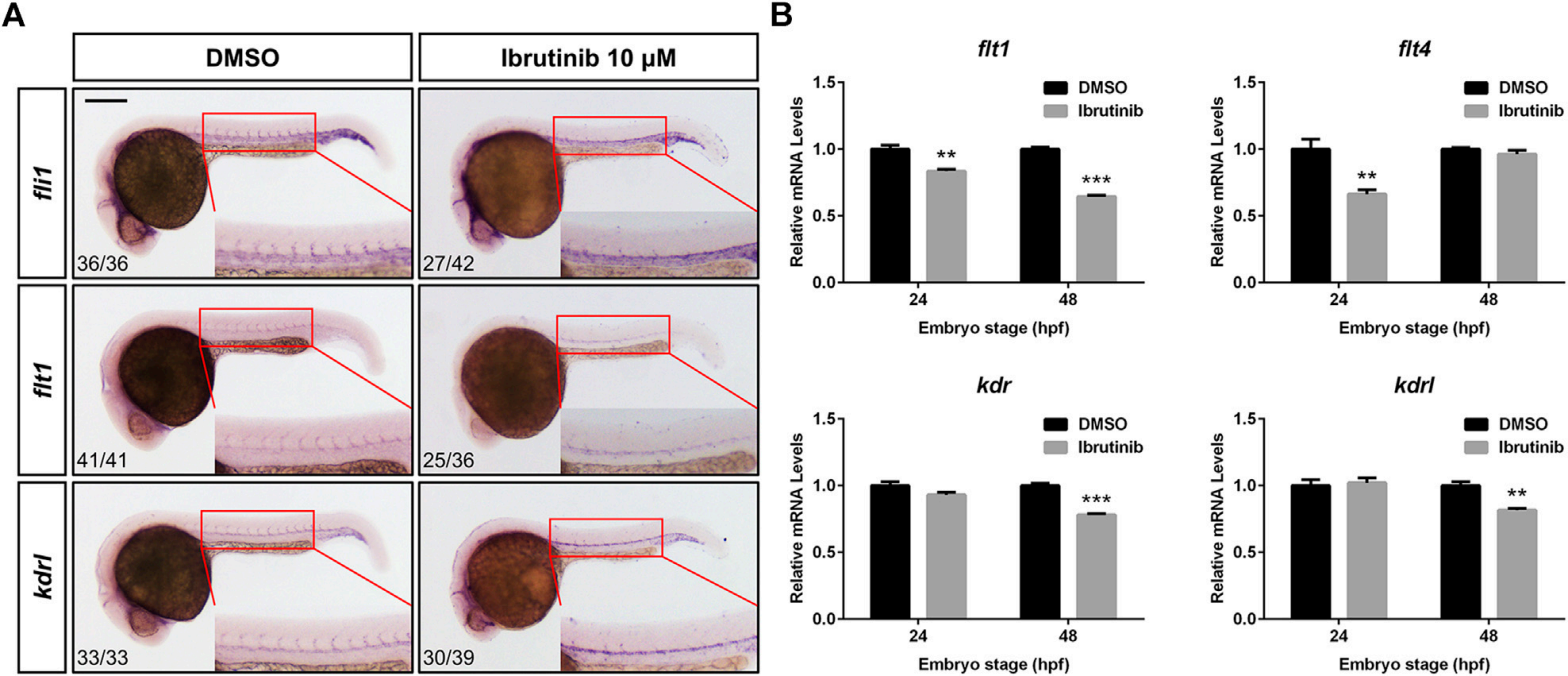
Ibrutinib exposure altered the expression of vascular development-related genes. **(A)** The expression pattern of vascular marker genes, *fli1*, *flt1*, and *kdrl*, was examined in ibrutinib-treated embryos by whole-mount *in situ* hybridization (WISH) at 24 h post-fertilization (hpf). Numbers at bottom left indicate the number of embryos with similar staining pattern among all embryos examined (Fisher’s exact tests, *p* < 0.001). **(B)** The qRT-PCR results showed the expression of VEGFR genes, *flt1*, *flt4*, *kdr*, and *kdrl*, in ibrutinib-treated embryos at 24 and 48 hpf. Asterisks indicate significant differences (DMSO vs. ibrutinib), **p < 0.01, ***p < 0.001.

To explore how ibrutinib affects vascular development, we examined the expression levels of relative genes. The WISH of embryos (10 μM ibrutinib treated at 12 hpf) at 24 hpf showed that the expression of *Fli-1 proto-oncogene, ETS transcription factor* (*fli1*), *fms related receptor tyrosine kinase 1* (*flt1*, also known as *vegfr1*), and *kinase insert domain receptor-like* (*kdrl*, also known as *vegfr2a*) in sprouting ISVs was remarkably suppressed (Figure 5A). Interestingly, we observed that the expression of these genes was increased in the forming DA of the treated embryos (Figure 5A). Additionally, qRT-PCR was performed to assess the expression of more vascular development-related genes. After ibrutinib exposure, the expression of *flt1* was significantly decreased at both 24 and 48 hpf, while *flt4* (also known as *vegfr3*) was downregulated at 24 hpf but unaffected at 48 hpf (Figure 5B). In contrast, *kinase insert domain receptor* (*kdr*, also known as *vegfr2b*) and *kdrl* were normally expressed at 24 hpf but downregulated at 48 hpf (Figure 5B). However, the expression of genes from the VEGF family, including *vegfaa*, *vegfab*, *vegfc*, and *vegfd*, was unaffected in ibrutinib-treated embryos (Supplementary Figure S2). Taken together, these results indicated that ibrutinib exposure alters the expression of vascular development-related genes.

Because vascular formation was closely related to hematopoiesis during embryogenesis, we also checked hematopoiesis-related genes in ibrutinib-exposed embryos. WISH was performed to detect the expression of major hematopoietic lineage genes, such as *Spi-1 proto-oncogene b* (*spi1b*) and *GATA binding protein 1a* (*gata1a*) for primitive hematopoiesis, *v-myb avian myeloblastosis viral oncogene homolog* (*myb*) for hematopoietic stem cells, *lysozyme* (*lyz*) for granulocytes, *hemoglobin, alpha embryonic 1* (*hbae1*) for erythrocytes, and *recombination activating 1* (*rag1*) for lymphocytes. The expression of these markers did not show any obvious differences between DMSO-treated and ibrutinib-treated embryos (Supplementary Figure S3), suggesting that hematopoiesis was not affected by ibrutinib.

## Discussion

Ibrutinib is widely used in the treatment of patients with B cell malignancies due to its marked efficacy, high safety, and good tolerance in clinical testing (Brown, 2018). Therefore, studies of ibrutinib mainly focus on patients or animal disease models (Dubovsky et al., 2014; Woyach et al., 2014; Singh et al., 2018). However, because adverse effects have also been demonstrated (Paydas, 2019; Lasica and Tam, 2020), studies on the toxicity of ibrutinib in normal development are needed. In the present study, we investigated the toxicological effect of ibrutinib on embryonic development by using zebrafish as a model organism. The results indicate that ibrutinib has potent developmental toxicity on zebrafish embryos. Embryos treated with ibrutinib show obvious malformation (Figures 1A,B) and could not survive (Figure 1C). Since ibrutinib could bind to proteins in many important signaling pathways, including BTK, BMX, EGFR, ITK, and JAK3 (Liang et al., 2018; Kim, 2019), we speculate that ibrutinib might induce developmental deficiency by affecting some crucial signaling pathways during embryogenesis.

The vascular development of vertebrates is commonly subdivided into two different processes, vasculogenesis, whereby aortas and veins are formed, and angiogenesis, whereby new blood vessels sprout from pre-existing ones (Risau et al., 1988). In zebrafish, vasculogenesis commences at approximately 12 hpf, when angioblasts start migrating from the lateral plate mesoderm to the midline. Subsequently, angioblasts differentiate into arterial precursor cells and venous precursor cells and then form the DA and PCV, respectively, at the 22-somite stage (about 20 hpf). After major axial vessels are established, sprouting angiogenesis occurs from the DA at approximately 22 hpf. These sprouting vessels, which are the future ISVs, grow dorsally and ultimately reach the level of the dorsal neural tube to form the dorsal longitudinal anastomotic vessel (DLAV) by fusing with neighboring vessels at 28–30 hpf (Childs et al., 2002; Hogan and Schulte-Merker, 2017). In this study, embryos treated with ibrutinib at 12 hpf, just before the vasculogenesis initiation, could still form the DA and PCV (Figure 2A), implying that ibrutinib might not affect vasculogenesis. In contrast, the cerebral vasculature and the ISVs in the trunk were strongly impeded (Figures 2A,B), suggesting that angiogenesis was perturbed by ibrutinib. To further explore the time window of ibrutinib inhibiting angiogenesis, embryos were exposed at different stages. Angiogenesis was severely suppressed in embryos exposed with ibrutinib at 12 or 24 hpf as expected (Figures 2C,D), considering that ISV sprouting occurs at around 22 hpf. Interestingly, embryos exposed at 36 hpf, when the primary ISV network is established, also exhibited remarkable angiogenesis deficiency (Figures 2C,D), implying that ibrutinib not only inhibits the sprouting of ISVs but also impaired existing ISVs. Moreover, embryos exposed at 12 hpf showed intact ISV networks when ibrutinib was washed out at 24 hpf, or weakly perturbed angiogenesis when ibrutinib was washed out at 36 hpf (Figures 2C,D). According to these results, we reasoned that ibrutinib might influence single or multiple proteins which were constantly needed for the whole angiogenesis process. Once ibrutinib was removed, the angiogenesis process would recover even if it had been suppressed before. The target protein by which ibrutinib inhibits angiogenesis is not thought to be BTK, the primary target of ibrutinib, because a direct link between BTK and angiogenesis is rarely reported. Besides, we verified this supposition by treatment with spebrutinib, another well-known BTK inhibitor, as well as *btk* gene knockdown by MO in zebrafish embryos. The effect on angiogenesis was inappreciable in both experiments (Supplementary Figures S1A,B).

The formation and maintenance of blood vessel lumens by VECs are crucial for vascular function. In zebrafish, the DA begins to lumenize shortly after being assembled and forms a tube to allow blood flow at 24–26 hpf, followed by the lumenization of the PCV (Herbert et al., 2009; Ellertsdottir et al., 2010). In our study, the lumen size of the DA and PCV were both reduced in embryos treated with ibrutinib (Figure 3), implying that ibrutinib impaired vascular lumen maintenance. Blood circulation was prohibited in the treated embryos, as no erythrocytes were found in the collapsed blood vessels (Figure 3B). The morphology of VECs seemed to be normal in treated embryos (Figure 3C), thus we speculated that ibrutinib might affect the arrangement and junction of VECs to perturb vascular lumen maintenance. The proliferation and apoptosis of VECs were also examined in ibrutinib-exposed embryos. After treatment, the number of proliferated VECs was normal in the DA, PCV, and CV, but decreased in ISVs (Figures 4A,B), which was in line with the result that ibrutinib inhibited angiogenesis but not vasculogenesis. In contrast, apoptotic VECs were significantly increased in the DA, PCV, and CV, as well as ISVs (Figures 4C,D), suggesting that ibrutinib induced apoptosis of VECs, which might contribute to the impairment of lumen maintenance.

The VEGF/VEGFR signaling pathway is essential for multiple processes of vascular development and conserved among vertebrates. In zebrafish, the initial sprouting of angiogenesis occurs from the DA in a process dependent on the Vegfr2 paralogs, Kdr and Kdrl, as well as Vegfa, indicating that the Vegfa/Vegfr2 signals are critical for ISV formation (Covassin et al., 2006; Bahary et al., 2007). Flt1 also plays an important role in angiogenesis, because the knockdown of the *flt1* gene is reported to result in ISV hyperbranching (Krueger et al., 2011). Flt4 is highly expressed in the tip cells of sprouting blood vessels, and the Vegfc/Flt4 pathway is negatively regulated by Notch signaling to limit angiogenesis (Villefranc et al., 2013; Le Guen et al., 2014). A recent study reveals that zebrafish Vegfd genetically interacts with Kdr and Flt4 to modulate angiogenesis (Bower et al., 2017). As indicated by our WISH results, the expression of early vascular marker genes, *fli1*, *flt1*, and *kdrl*, was dramatically suppressed in the sprouting ISVs of ibrutinib-exposed embryos at 24 hpf (Figure 5A), confirming that the primary angiogenesis was inhibited. However, the WISH signals of these genes were increased in the developing DA (Figure 5A). Considering that the sprouting of VECs from the DA was impeded, we reasoned that these VECs were probably accumulated, which led to the increased number of VECs in the DA. The qRT-PCR results revealed that the expression of genes from the VEGFR family, *flt1*, *flt4*, *kdr*, and *kdrl*, was decreased in the ibrutinib-exposed embryos, but the fold changes were not as great as expected (about 0.6–0.9) (Figure 5B). Moreover, the expression of genes from the VEGF family, *vegfaa*, *vegfab*, *vegfc*, and *vegfd*, was unaffected by ibrutinib (Supplementary Figure S2).These results indicated that VEGF/VEGFR pathway might not be the only pathway influenced by ibrutinib to impair vascular development. The inhibitory activity of ibrutinib on vascular development was independent of hematopoiesis, as verified by the WISH examination of hematopoietic markers in the treated embryos (Supplementary Figure S3).

In conclusion, we have demonstrated that the BTK inhibitor, ibrutinib, has adverse effects on the vascular development of zebrafish embryos. Ibrutinib exhibited a potent inhibitory activity on angiogenesis, but not vasculogenesis, during embryonic development. We also showed that ibrutinib exposure led to the collapsing of the vascular lumen, as well as abated proliferation and excessive apoptosis of VECs. The expression alteration in vascular development-related genes suggests that ibrutinib might influence the VEGF/VEGFR signaling pathway. However, further study is still needed to identify the precise targets of ibrutinib for perturbing vascular development. To the best of our knowledge, this is the first demonstration that ibrutinib exposure exhibits vascular toxicity in an animal model, and could provide the theoretical basis for safety guidelines in clinical treatment using ibrutinib.

## Data Availability Statement

The raw data supporting the conclusions of this article will be made available by the authors, without undue reservation.

## Ethics Statement

The animal study was reviewed and approved by Ethical Committee of the Southern University of Science and Technology.

## Author Contributions

KW and HZ conceived and designed the project. KW and QX performed the experiments. KW wrote the manuscript.

## Funding

This work was supported by a grant from the National Natural Science Foundation of China (31601166 and 31771603) and the Shenzhen Science and Technology Program (JCYJ20170817104455478).

## Conflict of Interest

The authors declare that the research was conducted in the absence of any commercial or financial relationships that could be construed as a potential conflict of interest.

## **Abbreviations**

BTK, Bruton’s tyrosine kinase; CV, caudal vein; DA, dorsal aorta; hpf, hours post-fertilization; ISV, intersegmental vessel; MO, morpholino; PCV, posterior cardinal vein; VEC, vascular endothelial cell; VEGF, vascular endothelial growth factor; VEGFR, vascular endothelial growth factor receptor; WISH, whole-mount *in situ* hybridization.
