# Supplementary material for: The Bruton’s Tyrosine Kinase Inhibitor Ibrutinib Impairs the Vascular Development of Zebrafish Larvae
Source: Front Pharmacol. 2021 Jan 13;11:625498. doi: 10.3389/fphar.2020.625498 (PMC7838594; doi:10.3389/fphar.2020.625498)
Supplement: Supplementary file 1 [file datasheet1.docx]

Supplementary Material

# Supplementary Figures


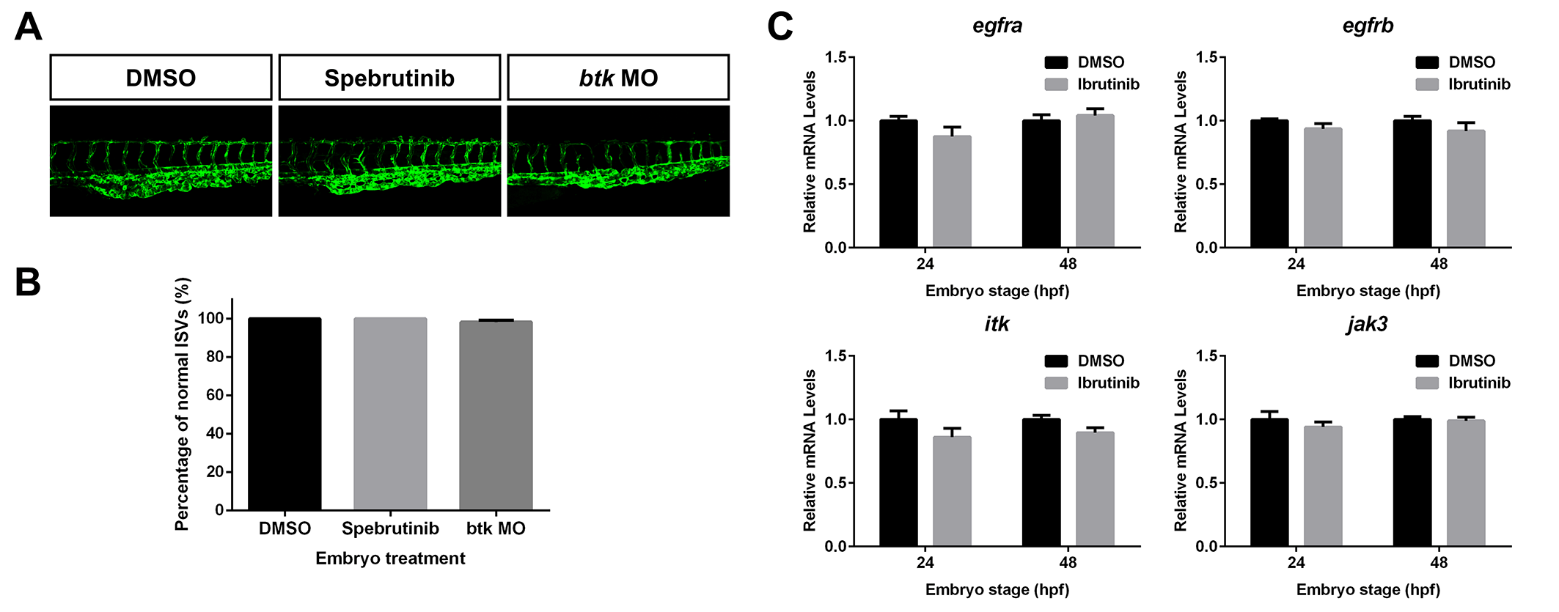


**Supplementary Figure 1.** Inhibiting or knockdown of BTK did not perturb vascular formation. **(A)** Confocal images of blood vessels in the trunk of *Tg*(*kdrl:EGFP*) embryos treated by spebrutinib or injected by *btk* MO. **(B)** The percentage of normal intersegmental vessels (ISVs) in embryos presented at **(A)**; *n* = 10 for each group. **(C)** The qRT-PCR results showed the expression of *egfra*, *egfrb*, *itk*, and *jak3*, in ibrutinib-treated embryos at 24 and 48 hours post-fertilization (hpf).


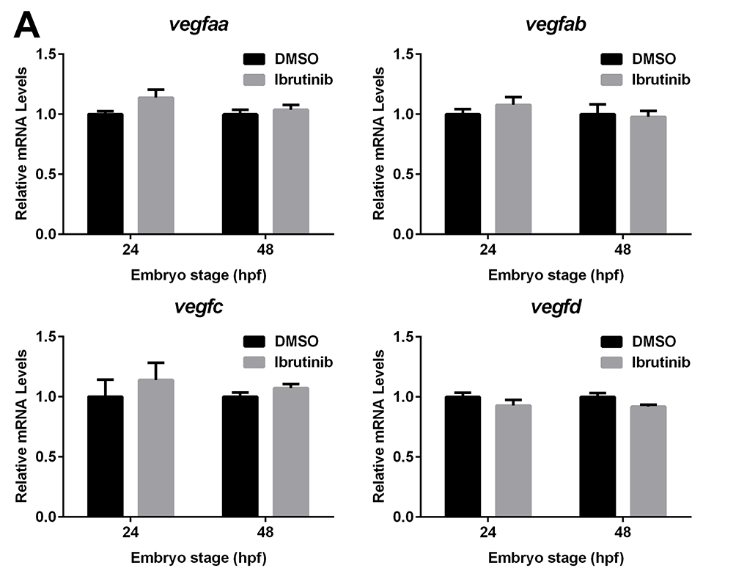


**Supplementary Figure 2.** Ibrutinib exposure did not change the expression of VEGF genes. **(A)** The qRT-PCR results showed the expression of *vegfaa*, *vegfab*, *vegfc*, and *vegfd*, in ibrutinib-treated embryos at 24 and 48 hours post-fertilization (hpf).


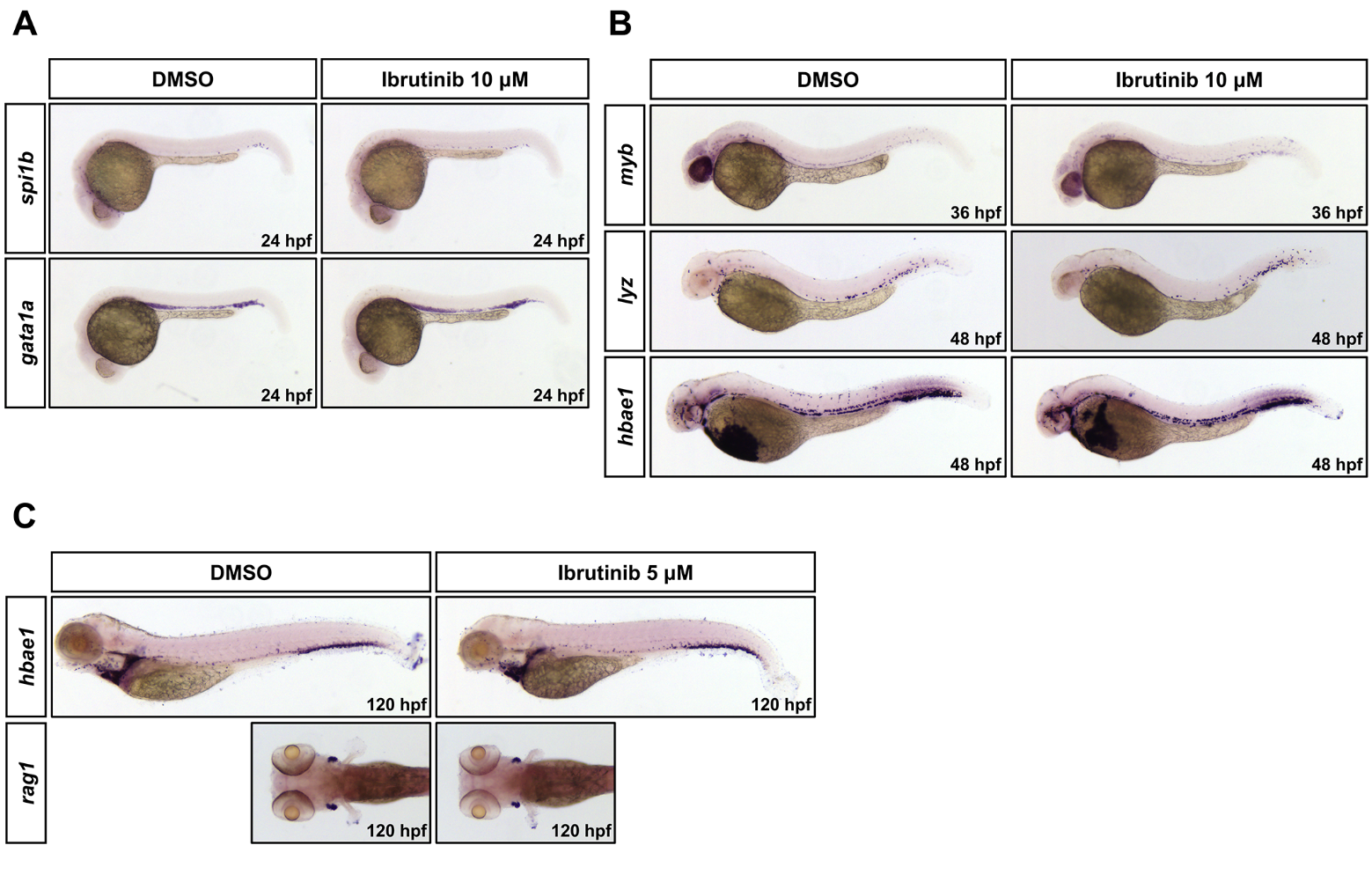


**Supplementary Figure 3.** Hematopoiesis was intact in ibrutinib-treated embryos. **(A-C)** The expression pattern of hematopoietic markers, *spi1b*, *gata1a*, *myb*, *lyz*, *hbae1*, and *rag1*, was examined in ibrutinib-treated embryos by whole-mount in situ hybridization (WISH) at respective stages.
